# State-Level Income Inequality and County-Level Social Capital in Relation to Individual-Level Depression in Middle-Aged Adults: A Lagged Multilevel Study

**DOI:** 10.3390/ijerph17155386

**Published:** 2020-07-27

**Authors:** Saloni Dev, Daniel Kim

**Affiliations:** Department of Health Sciences, Northeastern University, Boston, MA 02115, USA; dev.s@northeastern.edu

**Keywords:** depression, income inequality, social capital, United States

## Abstract

In the US, the incidence of depression and suicide have followed escalating trends over the past several years. These trends call for greater efforts towards identifying their underlying drivers and finding effective prevention strategies and treatments. One social determinant of health that plausibly influences the risk of depression is income inequality, the gap between the rich and poor. However, research on this association is still sparse. We used data from the National Longitudinal Survey of Youth 1979 and the US Census to investigate the multilevel lagged associations of state-level income inequality with the individual-level odds of depression in middle-aged adults, controlling for state- and individual-level factors. We also examined the independent associations of county-level social capital with depression and explored whether it mediated the income inequality relationship. Higher income inequality at the state level predicted higher odds of individual-level depression nearly 2 decades later [OR for middle vs. lowest tertile of income inequality = 1.35 (95% CI: 1.02, 1.76), OR for highest vs. lowest tertile = 1.34 (95% CI: 1.01, 1.78)]. This association was stronger among men than women. Furthermore, there was evidence that county-level social capital independently predicted depression and that it mediated the income inequality association. Overall, our findings suggest that policies attenuating levels of income inequality at the US state level and that leverage social capital may protect against one’s likelihood of developing depression.

## 1. Introduction

In 2017, 17.3 million adults in the US experienced at least one episode of major depression [1]. Depression is a leading cause of disability, mortality, and has been associated with other high-burden health conditions such as cardiovascular diseases [2,3,4]. Over the past decade, significant progress has been made towards time- and cost-effective psychotherapies and psychotropic medications to address a rising trend of depression. However, to further reduce the high burden of depression, there is a fundamental need to identify its root causes. Using a social determinants of health lens, identifying the role of societal conditions in influencing the risk of depression holds particular promise.

Social inequality, or the unequal distribution of valued resources, services, and positions within a society [5], has been identified as a major social determinant of common mental illnesses including depression [6]. One prominent component of social inequality is income inequality, i.e., the unequal distribution of income within a population [7]. In the US, income inequality has been growing over the past several decades, with the top 10 percent of the population averaging nearly nine times as much income as the bottom 90 percent [8]. While the relationship between absolute income and depression has been well established [6], research on the impact of relative income or income inequality on depression remains sparse.

Recent systematic reviews and meta-analyses point to a greater risk of depression among those living in areas with higher levels of income inequality, but report heterogeneous findings and small pooled effect sizes [9,10]. The latter may be the case for several reasons. First, previous studies to date have not accounted for the possible lagged associations of income inequality with health [11]. In the case of mental health, such lagged effects appear plausible since income inequality has been found to influence cognitive functioning among older adults nearly two decades later [12]. Second, as suggested by Chen and Crawford [13], income inequality systematically varies across geographic scales and thus, there is a need to further investigate the association at a specific geographic level, especially at larger, less homogeneous spatial levels such as the state level where income inequality may be more pronounced. Furthermore, income inequality has been posited to adversely impact health through eroding community social capital, including interpersonal trust and the structures that facilitate social interactions, and thereby promoting social isolation and loneliness [9,14,15]. Relatedly, there is a lack of empirical testing of the hypothesized mediating role of area-level social capital for the income inequality-depression relationship, including taking into account the temporal order between income inequality, social capital, and depression. Of the studies included in Patel et al. [9] and Ribeiro et al. [10], only seven studies were longitudinal, of which only three studies were based in the US [16,17,18]. While Pabayo, Kawachi, and Gilman [16] as well as Muntaner et al. [17] included a relatively short 2–3 year time lag, Fiscella and Franks [18] investigated a nearly decade long lag but measured income inequality at the primary sampling unit level. None of these studies tested for mediation of the income inequality associations by social capital.

In the present study, we investigated the multilevel lagged associations of state-level income inequality with individual-level depression among middle-aged adults in the contiguous US, controlling for state- and individual-level factors, with time lags of close to two decades. In addition, we explored whether county-level social capital mediated this relationship. In line with Wilkinson’s theory [19], we hypothesized that higher state-level income inequality would be associated with higher odds of depression at the individual level, and that this association would be mediated by county-level social capital.

## 2. Materials and Methods

### 2.1. Study Population

The National Longitudinal Survey of Youth 1979 (NLSY79) is an ongoing longitudinal study that follows a nationally-representative sample of youths born between 1957 and 1964 in the US and gathers information on labor market activities, health, and demographic and socioeconomic factors. The first survey was conducted in 1979 and included a sample of 12,686 individuals (14–22 years) selected through a multistage stratified area probability sample of dwelling units and group quarter units. These participants were interviewed annually until 1994 and bi-annually thereafter. Based on the knowledge that income inequality accrues over time [20] and the differences in the timelines of when Alaska and Hawaii were incorporated as US states, the study sample for our current analyses was limited to NLSY participants living in the 48 states of the contiguous US.

### 2.2. Outcome

For each NLSY79 participant, depression was measured using a previously validated subset of 7 items from the original 20-item Center for Epidemiological Studies Depression Scale (CES-D). Each item asked the respondent to self-report the frequency of occurrence of negative (e.g., poor appetite) and positive symptoms (e.g., feeling happy) over the preceding week on a 4-point Likert scale ranging from 0 (<1 day) to 3 (5–7 days). Internal consistency is relatively high for the scale (Cronbach’s α = 0.84). We analyzed depression as a dichotomous outcome using the validated cut-off score of 8, with total scores ranging from 0 to 21 [21]. If one item were missing, the scale scores was coded as missing by the data providers. Data on depression was obtained from the 50-and-over Health Module, administered over a number of years (between 2008 and 2014) when a NLSY79 participant turned 50 years of age. The mean score on the scale for the sample was 3.9 (SD = 4.53).

### 2.3. State-Level Income Inequality Measure

Income inequality was measured using the Gini coefficient at the state level. We selected the state level because at smaller geographic levels, populations are more homogenous and income inequality is less pronounced. The Gini coefficient represents the dispersion of income across the entire income distribution [22]. It is derived from the Lorenz curve, which displays the cumulative share of total income accruing to successive income intervals. It ranges from theoretical values of 0 (indicating perfect equality) to 1 (reflecting perfect inequality, where one individual receives all the income). In keeping with previous work [12], we assumed a relatively long lag period for the effects of income inequality on depression of roughly two decades, and used the state-level Gini coefficient from the 1990 US Census. We modeled it using state-based tertiles to allow for a potential non-linear relationship, while maximizing power as compared to a larger number of categories such as quartiles or deciles. States with a Gini coefficient less than 0.42 were categorized as having low income inequality, those with values between 0.421 and 0.438 were designated as having medium income inequality, and those with values higher than 0.438 were assigned as having high income inequality.

### 2.4. County-Level Social Capital Measure

Social capital is characterized by the level of civic participation and social interactions which can be measured by the density of formal and informal social organizations [23]. We used the social capital index developed by Rupasingha and colleagues [24] as a measure of county-level social capital. This index was initially created for the year 1990 using 13 variables including the total density of non-profit, social, civic, and religious organizations in a county, percentage of population that voted in presidential elections, and response rate to the decennial census. Principal component analysis was used to create a single index from these variables. This index was then updated for the years 1997, 2005, 2009 and 2014. For our analysis, we used data on the index corresponding to the year 1990 (as a main exposure) and 1997 (as a potential mediator). Social capital index for 1990 was categorized into three levels based on the cut-points used by Rupasingha et al. [24].

### 2.5. Individual-, County-, and State-Level Covariates

At the individual level, demographic and socioeconomic covariates gathered from the NLSY79 included age, sex, race/ethnicity, marital status in 1998, highest education obtained in 2008, and net family income in 1990. At the county and state levels, we controlled for median household income and the percentages of Blacks, Hispanics, and those 65 years or older in the year 1990. We rescaled median household income at the state and county levels to reflect a USD 10,000 unit change, and measures of racial/ethnic composition groups to represent a 5-percentage point unit change, except for the percentage of the population that was Hispanic at the state level, which was categorized into tertiles.

### 2.6. Statistical Analysis

To examine the lagged associations between state-level Gini coefficient and individual-level depression, we estimated multivariable logistic regression models, controlling for individual and state-level covariates, in both sexes combined (Model 1) and in men and women separately (Models 2 and 3, respectively). In sensitivity analyses, we assessed the robustness of the results after controlling for the change in the state of residence over the nearly 2-decade lag period. We further explored the association between county-level social capital for the year 1990 and individual-level depression, controlling for individual- and county-level covariates (Model 4). Last, we tested for possible mediation of the state-level income inequality association with depression by county-level social capital measured in 1997 using the 3-step Baron and Kenny approach [25]. The first step of this approach is reflected in Model 1. The second step is captured by a model that examines state-level income inequality as a predictor of county-level social capital (Model 5). The third step of this approach is tested in a model that adds county-level social capital to Model 1 (Model 6).

Of the total sample included in the current study, approximately 17% of participants had missing data on net family income and 5% were missing information on marital status as well as the highest education attained. To handle these missing data, we implemented a multiple imputation approach [26] under a missing at random assumption for missing values. Logistic regression models were independently applied to 10 copies of the data, each with missing values imputed. The analyses adjusted for clustering at the level of primary sampling unit and were weighted to correct for non-response and the probability of selection. All analyses were performed using SAS, version 9.4. (SAS, Cary, NC, USA) This study was approved for an exemption from human subjects by the Office of Human Subject Research Protection at Northeastern University. 

## 3. Results

Our analytic sample included 6997 individuals residing within the 48 contiguous US states. Sample characteristics are presented in Table 1. Slightly over half of the sample was male (52.6%), approximately 50% was non-Black and non-Hispanic, and the average age was 17.68 years at baseline (SD = 2.24). A majority of the sample was married in 1998 (58.24%), had at least a high school diploma or associate’s degree in 2008 (63.23%), and had a household income between USD 25,000 and USD 50,000 (37.25%). In total, 17.4% of the study sample reported having depression on the 50-and-over module.

Compared to those residing in states belonging to the lowest tertile of state-level income inequality, there was a higher odds of depression measured nearly two decades later among those living in states belonging to the middle tertile (OR = 1.35, 95% CI: 1.02, 1.76) and highest tertile (OR = 1.34, 95% CI: 1.01, 1.78; see, Table 2, Model 1). This association was stronger among men (OR for middle vs. lowest tertile = 1.93, 95% CI: 1.17, 3.16; OR for highest vs. lowest tertile = 1.71, 95% CI: 1.002, 2.91) than women (OR for middle vs. lowest tertile = 1.06, 95% CI: 0.76, 1.47; OR for highest vs. lowest tertile = 1.15, 95% CI: 0.83, 1.59; see, Table 2, Model 2 and 3). However, we found no evidence for a dose–response relationship between state-level income inequality and individual-level depression (*p* for trend = 0.08; Model 1). In sensitivity analyses that controlled for changes in the state of residence of each NLSY study participant over the nearly 20-year period, these results remained robust.

For county-level social capital, there was an inverse association with individual-level depression nearly two decades later (see, Table 2, Model 4). This association was observed when counties with the highest level of social capital were compared to those with the lowest level of social capital (OR for counties belonging to highest vs. lowest tertile = 0.73 (95% CI: 0.58, 0.93). In addition, we observed a significant linear trend in this relationship (*p* for trend = 0.02).

Last, we found evidence that the association between state-level Gini coefficient and individual-level depression was mediated by county-level social capital. Compared to states in the lowest tertile of income inequality, those in the highest tertile had 0.46 (*p* < 0.001; Table 2, Model 5) units lower social capital at the county level. Regressing individual-level depression status on state-level income inequality while also including county-level social capital simultaneously showed that the effect estimates for income inequality were no longer significant (OR for middle vs. lowest tertile = 1.32, 95% CI: 0.96, 1.82; OR for highest vs. lowest tertile = 1.26, 95% CI: 0.92, 1.73; Table 2, Model 6) while the effect estimates for social capital were significant (OR for highest vs. lowest tertile = 0.72, 95% CI: 0.53, 0.98; Table 2, Model 6).

## 4. Discussion

Our findings suggest that higher levels of income inequality at the US state level are linked to a higher odds of individual-level depression nearly 2 decades later. This association is stronger among men than women. In addition, we find that higher county-level social capital predicts a lower odds of individual-level depression, and find evidence that it mediates the state-level income inequality association.

Our study findings bolster the conclusions of recent meta-analyses that have reported a higher risk of depression in populations with greater income inequality [9,10]. In contrast to our study, the meta-analysis by Patel et al. [9] primarily included cross-sectional studies and only a small number of longitudinal multilevel studies, all with relatively short time lags (<10 years). Hence, our study contributes to the literature by providing empirical evidence to support a longer lagged association for income inequality. Moreover, our findings conflict with the findings from some previous studies that have reported no positive association, or even an inverse association between area-level income inequality and depression [27,28,29]. Differences in the nature of the data used (i.e., non-lagged cross-sectional data in previous studies), the geographic unit of analysis, and the study setting may explain the discrepancies in findings.

Sex differences in the association between income inequality and depression have been reported previously at the state level [16]. Contrary to our findings, Pabayo and colleagues [16] observed a positive association only among women and not men. The cross-sectional setting of that study could play a role in the discrepancy; plausibly, income inequality may be more detrimental to men’s mental health due to genetic make-up [30,31] or stereotypical gender and social roles [32], especially over a longer period of time. On the other hand, states with high income inequality may have other laws that compensate for the harmful effects of income inequality among women after several years of exposure. For example, studies have found that state-level policies concerned with reproductive rights and economic autonomy may have a protective effect on women’s mental health [33,34]. Further investigation at the intersection of laws and policies that foster income inequality and those that facilitate mental health and well-being across sexes are imperative to inform actions.

Furthermore, our results lend support to the previously reported role of social capital in mediating the association between income inequality and depression [23,35,36], and contributes towards our understanding of the potential mechanisms underlying the observed income inequality association at the state level. Specifically, higher income inequality at the state level is likely to foster underinvestment in social goods, unequal access to public resources, latent social conflict, relative deprivation, and erosion of social bonds [23]. Whereas income inequality was measured at a higher geographic level, social capital at the county level might reflect greater political participation and the development of social and non-profit organizations locally, fostering network ties and social support. It may also offer individuals a sense of social integration and fairness. As reported in our findings, social capital has also been found to have protective associations with depression in other studies [37].

### 4.1. Strengths and Limitations

To our knowledge, this is the first study to investigate the lagged associations of state-level income inequality with individual-level depression in the United States, including an assessment of social capital as a potential mediator. The temporal order of our measures reduced the risk of reverse causation. Our study also attempted to address the lack of methodological homogeneity among studies on this association, as identified in recent meta-analyses [9,10], by including measures that have been widely used in previous studies. Finally, the use of data from a nationally-representative sample adds to the generalizability of our findings.

Nonetheless, there are several limitations to our study. First, we used the Gini coefficient to measure income inequality, but this measure has been critiqueded for its weaknesses. For example, due to the properties of the Lorenz curve, two states with different patterns of income inequality can have similar Gini coefficient values [38]. Other income inequality measures such as income percentile ratios and the Atkinson Index have been argued to offer more nuanced measures. Nonetheless, the Gini coefficient is the most commonly used measure of income inequality. Second, only about 7500 of the 12,868 original participants at baseline in 1979 had data from the 50-and-over module between 2008 and 2013. Hence, our analytic sample might be subject to selection bias due to differences in associations in those who either dropped out or were lost to follow up after baseline. Third, our lagged cross-sectional study design involved a long time period between the exposure and outcome of interest. Since changes in the covariates were not taken into account, we cannot rule out the possibility of residual confounding by time-varying factors. Last, the generalizability of our findings does not necessarily extend to younger or older adult populations, and should be explored in future studies. 

### 4.2. Policy Implications and Future Directions

In light of America’s current suicide epidemic [39], our findings offer new evidence linking income inequality to depression that should be informative to researchers, mental health professionals, and policy makers. Specifically, the results presented here have important implications for contemporary policies informing the distribution of income in societies for reducing income inequality and preventing the worsening of mental health among Americans. Policy makers should consider supporting policy initiatives (e.g., tax reform policies) that can reduce state-level income inequality, and should extend their support in investments for the prevention and treatment of depression. In future, more research is needed to confirm the causal nature of these relationships, delineate the specific causal pathways linking income inequality to depression, and determine the subpopulations at greatest risk. Finally, mental health researchers and professionals should target such pathways and provide effective treatments to individuals in settings with high levels of income inequality.

## 5. Conclusions

In a sample of middle-aged adults in the contiguous US, we find that living in a state with a higher level of income inequality was associated with a higher odds of depression, and evidence that this association is mediated by social capital at the county level. Our findings call for a further exploration of the underlying pathways for these associations, and generate new support for policies that can reduce levels of income inequality at the state level to address the growing burden of depression among Americans.

## Figures and Tables

**Table 1:** Sample Characteristics (*n* = 6997).

Variable	*n* (%) or Mean (SD)
*Individual Level*	
*Sex (1979; n = 6997)*	
Female	3319 (47.4%)
Male	3678 (52.6%)
*Race/Ethnicity (1979; n = 6997)*	
Non-Black, Non-Hispanic	3521 (50.32%)
Non-Black, Hispanic	1332 (19.04%)
Black	2144 (30.64%)
*Marital Status (1998; n = 6635)*	
Never Married	1401 (21.12%)
Married	3864 (58.24%)
Separated	370 (5.58%)
Divorced	946 (14.26%)
Widowed	54 (0.81%)
*Highest Educational Attainment (2008; n = 6623)*	
None	890 (13.44%)
High School Diploma/Associate	4188 (63.23%)
Undergraduate, Graduate, or Professional Degree	1379 (20.82%)
Other	166 (2.51%)
*Net Family Income (1990; n = 5831)*	
≤USD 10,000	893 (15.31%)
USD 10,001–USD 25,000	1761 (30.20%)
USD 25,001–USD 50,000	2172 (37.25%)
USD 50,001–USD 100,000	876 (15.02%)
>USD 100,000	129 (2.21%)
*Depression (CES-D Scores)*	
<8	5781 (82.62%)
≥8	1216 (17.38%)
Age (in years; 1979; *n* = 6997)	17.68 (2.24)
***County Level***	
Social Capital (1990; *n* = 6997)	−0.45 (1.15)
Social Capital (1997; *n* = 6997)	−0.35 (1.06)
Median Household Income (1990; *n* = 6925)	USD 27,112.69 (8720)
Percentage Black Population (1990; *n* = 6894)	14.68% (14.04%)
Percentage Hispanic Population (1990; *n* = 6822)	10.67% (15.5%)
Percentage 65 years or older Population (1990; *n* = 6894)	12.47% (3.3%)
***State Level***	
Gini Coefficient (*n* = 6997)	0.44 (0.016)
Median Household Income (1990; *n* = 6997)	USD 54,756.07 (8655)
Percentage Black Population (1990; *n* = 6997)	12.89% (7.6%)
Percentage Hispanic Population (1990; *n* = 6997)	9.26% (9.8%)
Percentage 65 years or older Population (1990; *n* = 6997)	12.45% (2.1%)

**Table 2:** Coefficient estimates from multivariable logistic regression analysis of state-level income inequality, county-level social capital, and individual-level depression.

	Model 1 (Both Sexes) Outcome: Depression *n* = 6997	Model 2 (Women) Outcome: Depression *n* = 3678	Model 3 (Men) Outcome: Depression *n* = 3319	Model 4 (Both Sexes) Outcome: Depression *n* = 6997	Model 5 (Both Sexes) Outcome: Social Capital (1997) *n* = 6997	Model 6 (Both Sexes) Outcome: Depression *n* = 6997
	OR (95% CI)	OR (95% CI)	OR (95% CI)	OR (95% CI)	β (*p*-Value)	OR (95% CI)
*Gini Coefficient (State level; 1990)*						
0–0.42	Reference	Reference	Reference	-	Reference	Reference
0.421–0.438	1.35 (1.02, 1.76)	1.06 (0.76, 1.47)	1.93 (1.17, 3.16)	-	−0.22 (0.09)	1.32 (0.96, 1.82)
>0.438	1.34 (1.01, 1.78)	1.15 (0.83, 1.59)	1.71 (1.002, 2.91)	-	−0.46 (0.0009)	1.26 (0.92, 1.73)
*p for Trend*	*0.08*	*-*	*-*	*-*	*-*	*-*
*Social Capital (County level; 1990)*						
≤0	-	-	-	Reference	-	Reference
0.0001–1	-			0.85 (0.71, 1.03)	-	0.81 (0.66, 1.002)
>1	-			0.73 (0.58, 0.93)	-	0.72 (0.53, 0.98)
*p for Trend*	*-*	*-*	*-*	*0.02*	*-*	*-*
*Individual-Level Covariates*						
*Age*	1.04 (1.004, 1.08)	1.03 (0.99, 1.08)	1.06 (1.006, 1.12)	1.04 (1.003, 1.08)	−0.0009 (0.76)	1.04 (1.004, 1.08)
*Sex*		-	-			
Male	Reference			Reference	Reference	Reference
Female	1.67 (1.41, 1.97)			1.68 (1.42, 1.99)	−0.012 (0.41)	1.68 (1.41, 1.99)
*Race/Ethnicity*						
Non Black, Non Hispanic	Reference	Reference	Reference	Reference	Reference	Reference
Hispanic	0.72 (0.58, 0.90)	0.65 (0.50, 0.86)	0.80 (0.56, 1.15)	0.73 (0.55, 0.96)	0.004 (0.92)	0.73 (0.56, 0.96)
Black	0.81 (0.67, 0.97)	0.83 (0.65, 1.06)	0.81 (0.61, 1.08)	0.78 (0.63, 0.96)	0.008 (0.86)	0.78 (0.63, 0.96)
*Marital Status*						
Never Married	Reference	Reference	Reference	Reference	Reference	Reference
Married	0.85 (0.69, 1.04)	0.91 (0.68, 1.21)	0.80 (0.58, 1.06)	0.85 (0.70, 1.05)	−0.002 (0.94)	0.85 (0.69, 1.05)
Separated	1.85 (1.30, 2.64)	1.81 (1.14, 2.87)	2.14 (1.15, 3.98)	1.89 (1.29, 2.64)	−0.04 (0.27)	1.84 (1.28, 2.64)
Divorced	1.27 (0,98, 1.60)	1.31 (0.96, 1.81)	1.27 (0.84, 1.92)	1.26 (0.99, 1.61)	−0.03 (0.34)	1.26 (0.98, 1.61)
Widowed	0.63 (0.28, 1.45)	0.66 (0.25, 1.78)	0.75 (0.16, 3.58)	0.62 (0.26, 1.45)	−0.04 (0.63)	0.63 (0.26, 1.61)
*Highest Educational Attainment*						
None	Reference	Reference	Reference	Reference	Reference	Reference
High School Diploma/Associate	0.46 (0.37, 0.57)	0.46 (0.33, 0.63)	0.46 (0.32, 0.67)	0.47 (0.37, 0.59)	0.08 (0.003)	0.47 (0.38, 0.58)
Undergraduate, Graduate, or Professional Degree	0.27 (0.20, 0.36)	0.28 (0.19, 0.41)	0.26 (0.16, 0.43)	0.27 (0.21, 0.36)	0.21 (<0.0001)	0.28 (0.21, 0.37)
Other	0.58 (0.35, 0.97)	0.45 (0.28, 0.88)	0.85 (0.41, 1.76)	0.59 (0.35, 0.98)	0.18 (0.004)	0.60 (0.36, 0.99)
*Net Family Income*						
≤USD 10,000	Reference	Reference	Reference	Reference	Reference	Reference
USD 10,001–USD 25,000	0.66 (0.51, 0.85)	0.61 (0.46, 0.81)	0.74 (0.48, 1.15)	0.66 (0.51, 0.85)	−0.02 (0.48)	0.67 (0.52, 0.87)
USD 25,001–USD 50,000	0.47 (0.36, 0.61)	0.49 (0.36, 0.64)	0.44 (0.26, 0.72)	0.47 (0.36, 0.61)	−0.03 (0.33)	0.47 (0.36, 0.61)
USD 50,001–USD 100,000	0.39 (0.28, 0.55)	0.41 (0.28, 0.60)	0.37 (0.19, 0.71)	0.38 (0.27, 0.54)	−0.08 (0.39)	0.38 (0.27, 0.53)
>USD 100,000	0.38 (0.20, 0.74)	0.29 (0.12, 0.75)	0.49 (0.18, 1.32)	0.39 (0.19, 0.72)	−0.02 (0.54)	0.39 (0.21, 0.77)
***County-Level Covariates***						
Median Household Income	-	-	-	1.02 (0.92, 1.15)	−0.06 (0.82)	1.07 (0.94. 1.21)
% Black Population	-	-	-	0.99 (0.97, 1.03)	−0.008 (0.43)	1.02 (0.98, 1.05)
% Hispanic Population	-	-	-	0.98 (0.94, 1.03)	−0.05 (<0.0001)	0.98 (0.94, 1.03)
% Age 65 and older	-	-	-	1.01 (0.90,1.14)	0.03 (0.39)	0.99 (0.87, 1.12)
***State-Level Covariates***						
Median Household Income	1.004 (0.89, 1.14)	1.03 (0.88, 1.19)	0.96 (0.77, 1.19)	-	0.007 (0.86)	0.96 (0.84, 1.10)
% Black Population	0.97 (0.92, 1.03)	0.98 (0.92, 1.05)	0.93 (0.83, 1.05)	-	−0.15 (<0.0001)	0.92 (0.86, 0.99)
*% Hispanic Population*				-		
0–1.2%	Reference	Reference	Reference		Reference	Reference
1.3–4.4%	0.87 (0.66, 1.15)	0.87 (0.62, 1.22)	0.90 (0.50, 1.62)		−0.01 (0.93)	0.93 (0.72, 1.22)
>4.4%	0.79 (0.61, 1.04)	0.62 (0.44, 0.88)	1.17 (0.68, 2.02)		−0.25 (0.06)	0.82 (0.63, 1.07)
% Age 65 and older	1.007 (0.81, 1.24)	0.86 (0.64, 1.16)	1.19 (0.88, 1.61)	-	0.01 (0.80)	0.99 (0.80, 1.24)

Models 1, 2, and 3 adjusted for individual age in 1979, sex, race/ethnicity, marital status in 1998, highest education achieved in 2008, and net family income in 1990. At the state level, models controlled for median household income, the percentages of Blacks, Hispanics, and the percentaged aged 65 years or older in 1990. Model 4 adjusted for the aforementioned individual-level covariates as well as the following county-level covariates for the year 1990: median household income, the percentages of Blacks, Hispanics, and those aged 65 years or older. Model 5 and 6 adjusted for all individual-, county-, and state-level covariates. Social capital was modeled as a continuous variable for Model 5.
